# Improvement in Selective Serotonin Reuptake Inhibitor-Associated Sexual Dysfunction With Buspirone: Examining the Evidence

**DOI:** 10.7759/cureus.57981

**Published:** 2024-04-10

**Authors:** Kyra Lipman, Holly Betterly, Mousa Botros

**Affiliations:** 1 Psychiatry and Behavioral Sciences, University of Miami Miller School of Medicine, Jackson Memorial Hospital, Miami, USA

**Keywords:** side effects, delayed ejaculation, sexual dysfunction, erectile dysfunction, selective serotonin reuptake inhibitor, buspirone, generalised anxiety disorder, psychiatry

## Abstract

Sexual dysfunction is a common problem for patients taking antidepressants, with the highest prevalence rates observed with selective serotonin reuptake inhibitors (SSRIs). Sexual dysfunction can be distressing for patients and may lead to medication non-adherence; thus, it is important for the prescribers to be aware of the available treatment strategies, as well as of the strength of the evidence that supports their use. We present the case of a patient who developed delayed ejaculation after the initiation of sertraline for the treatment of depression. The patient’s sexual dysfunction resolved after the addition of buspirone. A discussion of this case is followed by a review of the existing literature examining the possible role of buspirone in the treatment of SSRI-induced sexual dysfunction.

## Introduction

Sexual dysfunction is a frequently encountered problem among patients taking antidepressant medications [1]. The prevalence of treatment-emergent sexual dysfunction ranges from 25 to 80% and is likely underreported [1]. A higher prevalence has been observed when patients are asked directly about sexual dysfunction as opposed to relying upon spontaneous self-report [1]. While sexual dysfunction has been observed with the use of almost all antidepressants, the highest rates have been reported with selective serotonin reuptake inhibitors (SSRIs) [2]. Sexual dysfunction can have a significant negative impact on quality of life and interpersonal relationships, which may lead to medication nonadherence [2]. It is crucial for psychiatrists to actively screen for sexual dysfunction in patients taking antidepressants, as well as to critically examine the supporting evidence for available treatment strategies. We present the case of a patient who experienced sexual dysfunction which emerged following the initiation of sertraline and resolved after the addition of buspirone. This is followed by a review of the existing evidence for the use of buspirone in the treatment of antidepressant-induced sexual dysfunction.

## Case presentation

The patient is a man in his mid-twenties with no significant past medical history and a past psychiatric history of schizophrenia, major depressive disorder, and panic attacks who was admitted to a residential forensic psychiatric unit for restoration of competency to stand trial. On admission, he reported depressive symptoms. Given a reported history of prior efficacy in the outpatient setting, sertraline was initiated and gradually titrated to 75 mg daily. Over the next few days, the patient was observed to be guarded, isolative, internally preoccupied, and paranoid, with a clinical presentation overall concerning for psychosis; risperidone was thus added and titrated to 3 mg twice daily.

On day 14, the patient reported experiencing new-onset delayed ejaculation, which he found bothersome. A serum prolactin level was checked and found to be elevated at 42 ng/ml, suggesting a potential contribution of risperidone to sexual dysfunction. A head CT was unremarkable. At this time, no changes were made to the dose of risperidone, as the patient's presentation of delayed ejaculation without any other symptoms of hyperprolactinemia was less consistent with risperidone-induced sexual dysfunction. Due to concern that sexual dysfunction may be secondary to sertraline, the dose was decreased to 50 mg daily. The patient did not report any improvement or change in sexual dysfunction after this dose reduction.

On day 20, the patient reported new-onset anxiety concerning for akathisia; thus, risperidone was discontinued, and he was instead started on haloperidol 5 mg twice daily, as well as benztropine 0.5 mg twice daily for the prevention of extrapyramidal side effects. Buspirone was also added to target sexual dysfunction and anxiety, with the dose titrated to 10 mg thrice daily. Sertraline was further uptitrated to 150 mg daily to target worsening anxiety, with this decision guided by a history of prior efficacy, an absence of clinical response at the present lower dose, and a lack of improvement in sexual dysfunction after the reduction in dose. On day 25, mirtazapine 15 mg was added at bedtime to target insomnia. 

On day 34, the patient reported an improvement in anxiety and depressive symptoms. He displayed a brighter affect, reduced anxiety, and increased engagement in activities on the unit. He reported some urinary hesitancy, which was attributed to the anticholinergic burden, at which point benztropine was discontinued. Haloperidol was also discontinued and replaced by quetiapine 300 mg at bedtime to target both psychosis and anxiety. Buspirone was able to be reduced to 5 mg thrice daily without re-emergence of sexual dysfunction nor worsening of anxiety.

**Figure 1:**
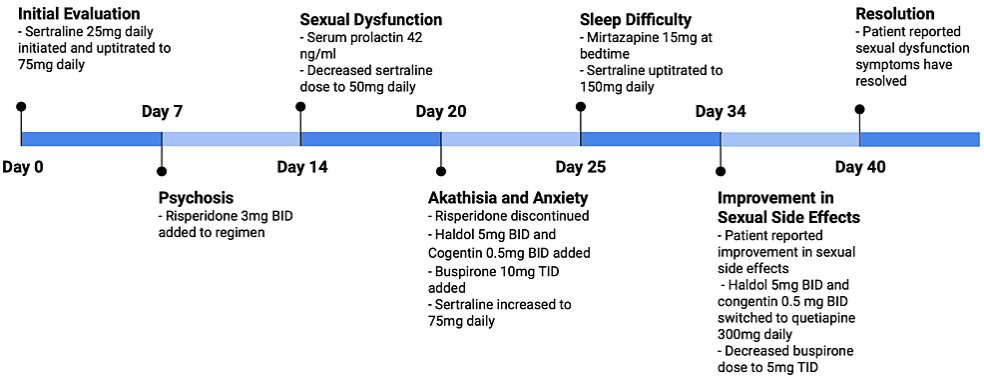
Timeline of Events

By hospital day 40, the patient reported that the delayed ejaculation had resolved. He demonstrated a return to his clinical baseline, tolerating his psychotropic regimen with a good clinical response and without adverse effects. A timeline of the patient's hospital course is outlined in Figure 1.

## Discussion

**Figure 2:**
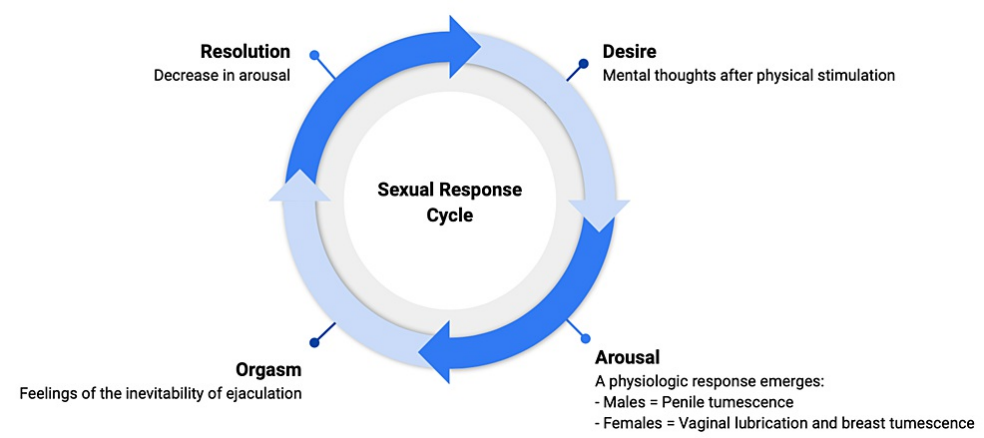
Normal Sexual Response Cycle

SSRI-induced sexual dysfunction may impact any phase of the sexual response cycle, which incorporates desire, arousal, orgasm, and resolution (Figure 2) [2-5]. Patients may report decreased libido (desire), decreased vaginal lubrication, inability to maintain an erection, and/or undesired changes in the timing of ejaculation [2-5]. One prospective study by Stahl et al. found that the incidence of sexual side effects was greatest with paroxetine, followed by fluvoxamine, sertraline, and fluoxetine [3]. Among SSRIs, paroxetine use has been correlated with the highest rates of delayed ejaculation, diminished desire, inability to maintain an erection in males, and insufficient lubrication in females [3]. However, one study by Modell et al. found that fluoxetine, paroxetine, and sertraline were associated with equal reductions in sexual desire, arousal, duration of orgasm, and intensity of orgasm in comparison to the patients’ baseline functioning [4].

Buspirone is a medication approved for the treatment of anxiety disorders, as well as for the short-term relief of anxiety. It exhibits a strong affinity for serotonin 5HT1a receptors, where it functions as a partial agonist. However, the precise mechanism through which it induces a clinical anxiolytic effect remains uncertain. Buspirone also demonstrates a moderate affinity for dopamine D2 receptors, where it displays weak antagonism [6,7]. It is rapidly absorbed, reaching peak plasma concentration within 90 minutes [7]. Metabolism occurs via oxidation by CYP 3A4, which produces an active metabolite, 1-pyrimidinylpiperazine. The elimination half-life is approximately 2 to 3 hours, with excretion primarily through the urine. The initial recommended total daily dose for the treatment of generalized anxiety disorder is 15 mg by mouth, with a therapeutic effect typically seen at higher doses of 20 to 30 mg daily. However, doses up to a maximum of 60 mg daily can be used. The most common adverse effect is dizziness. The potential for abuse is low, as is toxicity; there have been no reported fatalities in overdose [7].

In routine clinical practice, buspirone is used off-label as an adjunctive treatment to mitigate antidepressant treatment-emergent sexual dysfunction [7]. However, the available literature to support this practice is mixed. As early as 1987, it had been suggested that buspirone may improve sexual dysfunction in patients with generalized anxiety disorder [8,9], and by the following decade, a potential role for the mitigation of SSRI-associated sexual dysfunction had been proposed [8,10]. A 1999 study by Landen et al. re-analyzed data from a placebo-controlled trial initially designed to assess the efficacy of buspirone as an adjunct treatment in SSRI-refractory depression [8]. The reanalysis aimed to examine the potential role of buspirone in alleviating sexual dysfunction [8]. In addition to their existing regimen of either citalopram or paroxetine, the study participants were assigned to receive either adjunct flexible-dose buspirone (from 20 mg to 60 mg daily) or placebo. After four weeks, 58% of the buspirone group reported improvement in sexual dysfunction, versus only 30% of the placebo group. This difference was more pronounced in women. The response was seen within the first week of treatment, with no further improvements observed. The authors suggested that the effect seen in the buspirone group was likely secondary to antagonism of SSRI-induced sexual dysfunction rather than to augmentation of the antidepressant effect. The presumed mechanism for the improvement in sexual function was antagonism of the alpha 2 receptor by buspirone’s active metabolite; however, the role of the 5HT-1a agonism, or dopamine, could not be ruled out [8]. 

While the results of this study appear promising, those from a more recent randomized placebo-controlled trial by Michelson et al. are less so [11]. This study examined women who developed treatment-emergent sexual dysfunction after successful treatment with fluoxetine for a minimum of eight weeks. The participants were assigned to receive adjunctive treatment with either buspirone, amantadine, or placebo for four weeks. Sexual dysfunction improved across the board, with no statistically significant difference observed between groups. The authors concluded that buspirone and amantadine showed no greater efficacy than placebo for SSRI-associated sexual dysfunction [11].

In our patient’s case, delayed ejaculation was reported after the initiation of sertraline. It did not respond to a reduction in the dose of sertraline but did resolve after the addition of buspirone, and did not recur after subsequent uptitration of sertraline. While it is possible that buspirone may explain the alleviation of our patient’s sexual dysfunction, there are many other potential contributing factors that must be taken into consideration. Our patient was initially prescribed risperidone, which carries a risk of sexual dysfunction; thus, it is possible that its discontinuation may have contributed to improvement. The patient's serum prolactin level was elevated, which also provided some support for risperidone-induced sexual dysfunction. However, the overwhelming majority of male sexual side effects secondary to risperidone are decreased libido and erectile dysfunction, with limited reports of delayed ejaculation [12]. Furthermore, no other symptoms related to hyperprolactinemia were observed in our patient. Given this, risperidone-induced sexual dysfunction was considered a less likely cause of the patient's delayed ejaculation. There was no evidence of prolactinoma on head imaging, the presence of which was also initially considered as a possible explanation. As mentioned in the study by Michelson et al., placebo effects may contribute to the response in SSRI-associated sexual dysfunction, which we cannot rule out in our patient’s case [11]. We had discussed with our patient that the aim of adding buspirone was to address his sexual dysfunction, which may have predisposed him to anticipate such a response. Additionally, our patient was also prescribed mirtazapine, for which there is some evidence of efficacy as a treatment for SSRI-associated sexual dysfunction. In a retrospective study conducted by Atmaca et al., 20 patients receiving SSRI monotherapy for major depressive disorder displayed a statistically significant improvement in SSRI-induced sexual dysfunction after the addition of low-dose mirtazapine (15-45 mg daily) as adjunctive therapy [13]. However, improvement in symptoms was not observed until weeks 4 and 8, at which point clinical assessments were conducted. Our patient experienced an improvement in sexual dysfunction within 10 days of the initiation of mirtazapine, which is earlier than the timeframe observed in the study [13]. While we cannot say with certainty that mirtazapine had no role in providing some relief to the patient's sexual side effects, it is important to note that further investigation is warranted to determine the exact contribution of mirtazapine in this case, especially given the rapid improvement observed.

## Conclusions

The existing literature on the role of buspirone in SSRI-induced sexual dysfunction presents mixed findings. Buspirone may be responsible for our patient's improvement, but establishing causality is complex given the possibility of contribution from other factors. Prescribers should be mindful that correlation does not imply causation, and in our patient’s case, discerning the extent to which different factors may have contributed to the patient’s relief is challenging. Prescribers should rely on the evidence to guide their treatment decisions while concurrently considering the unique characteristics and individualized needs of their patients. Our case supports the need for additional research and close examination of the potential role of buspirone in improving SSRI-induced sexual dysfunction.
